# The impact of measurement based care at scale: examining the effects of implementation on patient outcomes and provider behaviors

**DOI:** 10.3389/frhs.2025.1659238

**Published:** 2025-11-28

**Authors:** Nicholas R. Forand, Jasmine Nettiksimmons, Amanda Brownell, Margaret T. Anton, Raven Truxson, Brandn Green, Colleen Marshall

**Affiliations:** 1 Two Chairs, San Francisco, CA, United States; 2 The Donald and Barbara Zucker School of Medicine at Hofstra/Northwell, Hempstead, NY, United States; 3 JG Research and Evaluation, Boseman, MT, United States

**Keywords:** measurement-based care, implementation, training, quality improvement, sustainment, depression, anxiety, psychotherapy outcomes

## Abstract

**Introduction:**

Measurement-based care (MBC) is an evidence-based practice; however there are challenges associated with implementing and sustaining this practice in care. This study examined the outcomes of an organization-wide implementation of MBC in a technology-supported psychotherapy practice. Outcomes were patient symptom change, clinician behaviors, and clinician performance.

**Methods:**

A total of *n* = 18,721 patients and 755 clinicians were included in the 6-month implementation. Change efforts targeted organizational alignment, technology integration, education and support, and cultural and operational change. Outcomes were assessed across three phases: pre-implementation, implementation, and post-implementation. Primary outcome measures for patients were percent change on the PHQ-9 and GAD-7. Estimates of differences between phases of implementation were computed using linear mixed effects models, adjusted for patient characteristics. Clinician behaviors associated with MBC were extracted from progress notes. Changes in individual clinician performance were assessed for clinicians with sufficient data across the implementation phases.

**Results:**

Patient outcomes improved significantly from pre- to post-implementation by approximately 5 percentage points for all outcomes. This represents a relative improvement of 23.5% on a combined PHQ-9 and GAD-7 measure. Clinicians demonstrated significant increases in MBC-related documentation behaviors. Among clinicians with sufficient data, 95% showed evidence of improved performance. Notably, clinicians whose baseline performance was superior showed greater improvements in performance.

**Discussion:**

Overall, this study suggests that structured MBC implementation was associated with improved patient outcomes, clinician behavior change, and clinician performance, although causal attributions are not possible given the retrospective non-randomized design. These results have implications for scalable implementation approaches in regular practice settings.

## 1 Introduction

### 1.1 Introduction to measurement based care

Measurement-based care (MBC), also known as routine outcome monitoring (ROM), is an evidence-based practice (EBP) that has been associated with improved outcomes for individuals with mental health conditions (1). MBC involves the systematic collection of clinical data throughout treatment to guide clinical decision-making and adapt treatment strategies (2). As an EBP, MBC has been endorsed by major professional organizations, including the American Psychology Association (APA) and the Substance Abuse and Mental Health Services (SAMHSA), for its role in enhancing treatment efficacy, patient engagement, and overall care quality (1, 2).

MBC follows a structured process where clinicians: (1) collect standardized assessments on patient symptoms, functioning, and treatment processes; (2) share this information with patients to enhance communication and engagement; and (3) adapt interventions based on insights from the patient-reported data (1, 3). This approach allows for personalized, responsive treatment, and reduces the risk of stagnation or deterioration in care (1, 4). Research indicates that MBC enhances therapy outcomes by enabling clinicians to identify and address care delivery and alliance challenges in real time, leading to reduced dropout, improved therapeutic alliance, and faster symptom resolution (5, 6).

### 1.2 Challenges and pitfalls in MBC implementation

Despite the benefits, implementation of MBC remains limited, with adoption rates among behavioral health providers estimated at less than 20% and only about 5% adhering to MBC following any evidence-based schedule (e.g., every session) (7). Various systemic barriers, including financial costs, time constraints, and the burden of ongoing monitoring may have contributed to the slow uptake of MBC in clinical practice (7, 8). One of the primary obstacles identified in the existing research literature on MBC implementation is the burden associated with the set up and maintenance of the practice (9). Establishing MBC infrastructure requires an investment in assessment tools, data management systems and clinician training, which can be costly, particularly for smaller clinics and community mental health providers (9, 10). Sustaining this practice requires consistent efforts from both patients and clinicians, which can be difficult in settings with high staff turnover, limited resources, or patient populations facing barriers to regular participation (3, 4).

Although strong evidence supports MBC as a gold standard practice, improper implementation or superficial adoption of MBC can negate its benefits and may even lead to adverse effects on both patients and clinicians (11). When routine assessment is mandated without effective training or support for clinicians, it can create negative experiences for both patients and clinicians (12). Administering measures without discussing their purpose or relevance can lead to decreased patient engagement, and lower therapeutic alliance and satisfaction with care (12). Clinicians may also experience frustration and burnout when required to collect data without proper training or guidance on how measurement should be integrated into care (12). Put simply, gathering data without integrating feedback into clinical care does not align with best practices in MBC, and may even be harmful (13). Conversely, patients who have been educated on the purpose of MBC and whose clinicians regularly discuss self-reported data in session report improved trust, self-awareness and strengthened therapeutic alliance (14).

The implementation science literature acknowledges the substantial set-up and maintenance costs incurred to install a new EBP, as well as the need for ongoing clinician support to drive the sustained clinical behavior change necessary before the impact of the EBP may be fully realized. Effective implementations often rely on resource and time-intensive interventions, including synchronous training, direct supervision, and individualized coaching of clinicians, which can limit the scalability of this approach (15). Time to outcomes can also be lengthy; authors caution that the initial installation of a new EBP typically takes 2–4 years (16). Furthermore, achieving *full, sustainable* implementation—to the point where it demonstrably improves patient outcomes—is a multi-stage process often cited as taking 3–7 years (15, 17). Understandably, leaders might be reluctant to invest in MBC without certainty about the impact or sustainability of the implementation within their organizations and the long timelines needed before impact might be observed (14).

Although MBC is a gold-standard EBP, the challenges associated with implementation, including effectiveness, scalability, and time to outcome, highlight the need for new models of implementation that can drive clinical behavior change efficiently and sustainably.

### 1.3 The current study

The extant literature reflects a gap in the science: there are few validated models for efficient, MBC implementation that are scalable and lead to improved patient outcomes on timelines that demonstrate and confirm impact. This study analyzes the patient and provider outcomes associated with MBC implementation in a real-world, large-scale, technology-enabled psychotherapy practice. MBC implementation occurred across the entire organization with a large, distributed, and remote workforce (755 teletherapy clinicians and over 18,000 patients). Implementation focused on motivating clinical behavior change on a short time scale, with the initial implementation intended to be completed within six months and observable impact on clinician behaviors and patient outcomes expected within one year (this is in contrast to the 3–7 year timeline cited in the literature). To achieve these goals, this model leveraged technology platforms to support the use and interpretation of outcome measures, provide feedback to clinicians, promote MBC adherence, and track and promote clinical behavior associated with MBC. Existing and new clinicians used learning platforms to support self-lead asynchronous training and to evaluate changes in their clinical skills. There was minimal direct oversight, supervision, or coaching in conjunction with self-directed learning. The technology suite for collecting, sharing, and providing clinical decision support to clinicians has been described in a previous manuscript (18). This study focuses on a pre-training vs. post-training comparison of patient treatment outcomes. The secondary outcomes of interest measure fidelity to the MBC model, and include indicators of training effectiveness and MBC-consistent clinician behaviors and performance.

## 2 Methods

### 2.1 Setting and context

The MBC implementation was conducted at Two Chairs, a hybrid technology-enabled behavioral health company founded in 2017 that provides psychotherapy to self-pay, commercially insured, and publicly insured individuals. During the period of data collection, services were available in California and Washington. Patients served by Two Chairs are aged 18 years or older and receive outpatient psychotherapy for anxiety, depression, and related conditions. All study procedures were deemed exempt by the Sterling institutional review board, Atlanta, Georgia, United States.

Two Chairs' clinical model allows clinicians to practice using their preferred theoretical orientation and approach. In 2021, MBC was selected as an EBP to implement because it can apply across different theoretical therapeutic approaches and clinical diagnoses (19, 20). During the initial implementation of MBC, the management team at Two Chairs launched a clinician-facing software platform and a set of automated patient-reported measures, the PHQ-9, GAD-7, Satisfaction with Life Scale, and a therapeutic alliance measure, accompanied by a brief self-led training in how to use these measures in care. Surveys measuring adherence to MBC were also implemented to generate a fidelity performance metric. In some ways this implementation was successful, in that it led to MBC survey completion rates of greater than 90%. However, anecdotal reports after the implementation suggested that clinicians had low buy-in, and there was also limited impact on patient outcomes, consistent with the known issues related to MBC implementation (19, 20). In light of this limited success, a quality improvement team at Two Chairs was tasked in late 2022 with understanding the problem and developing a quality improvement effort to strengthen the MBC implementation. The ultimate goal of the quality improvement effort was to improve clinical outcomes.

### 2.2 EPIS framework

The quality improvement team organized their approach around elements of the Exploration, Preparation, Implementation and Sustainment (EPIS) framework, a comprehensive model used to guide the integration of EBPs into real world service settings. EPIS consists of four phases, described as follows: (1) Exploration: identify unmet clinical or community needs, survey available EBPs to address these gaps, and identify whether an intervention aligns with their goals and resources; (2) Preparation: lay groundwork for successful implementation by assessing organizational readiness, developing strategies, and leveraging strengths; (3) Implementation: introduce the EBP, and iterate and refine based on feedback; and (4) Sustainment: the EBP is embedded into routine practice to ensure benefits are maintained and strengthened over time (21). The quality team was focused on identifying and leveraging known drivers of success, including clinical competency, leadership, and organizational factors, within and across EPIS phases (22).

### 2.3 Exploration

The quality improvement team began by identifying specific gaps in the initial implementation, including ineffective efforts at generating clinician buy-in and inadequate education, supervision, and support for clinical behavior change. Other gaps included inappropriate use of alliance scores as a clinician performance metric, low perceived clinical utility of some standardized measures, and inadequate clinical decision support. Several existing strengths were also identified including the robust software package, very high levels of MBC survey completion, and strong mission alignment across levels of the organization. Further details on the Two Chairs software package and outcomes can be found in a previous publication (18).

### 2.4 Preparation

#### 2.4.1 MBC implementation design

Two Chairs developed a formal plan based on the findings of the exploration period. Quality improvement team members shared evidence of the current training gaps and examples of best practice training with company leadership and linked improvement in clinical outcomes to company goals. Similar efforts were made to drive alignment among upper and lower levels of clinical management. The senior leader of the clinical organization served as an internal advocate of this work.

#### 2.4.2 Training development

A four-part training series was developed, each focused on a specific aspect of using MBC within Two Chairs' organizational context. Each self-led module took about 45 min to complete. Training focused on addressing: (1) the rationale and evidence for MBC and why the company chose to adopt it; (2) effective practices for using patient-reported data to guide care decisions; and (3) pragmatic information on how to interpret and use the standardized measure set.

The training modules were as follows:

- Module 1: MBC overview
- Module 2: Therapeutic alliance
- Module 3: The PHQ9 and GAD7
- Module 4: Putting it all together

#### 2.4.3 Skills assessment

After modules 2–4, clinicians participated in hour-long practice groups facilitated by the quality team focused on demonstration of the clinical skills they learned. After module 4, clinicians submitted two recordings to the quality team demonstrating: (1) how to introduce MBC to patients, and (2) how to respond to specific changes in symptoms. These recordings were rated by the quality team using a structured scoring system developed in-house. Clinicians who received non-passing scores were given coaching and required to submit new recordings.

#### 2.4.4 MBC system improvements

A set of technical adjustments were planned for the MBC system to improve functionality. These included the development and testing of a new measure to replace the Satisfaction with Life Scale and the adjustment of MBC system rules regarding alerts around meaningful symptom change. These changes were scoped and implemented across the training period in 2023 and into early 2024.

### 2.5 Implementation

The implementation of the training program began in May 2023 and continued until the end of 2023, with all training and skill assessment activities complete within six months. Additional support included dedicated time for training, skills practice, and manager evaluation of clinician skills. Two Chairs' employment model allowed for adequate and compensated time to be set aside for clinicians to complete required training and oversight. Completion of training exercises and skills assessments were tracked and if clinicians did not complete the required training they would be reminded, and eventually their managers would be informed. Training completion ranged from 75% to 79% across the four modules. The quality improvement team collected feedback from clinicians on training design and effectiveness and adjusted training approaches as the implementation progressed. The use of average alliance score as a performance metric was de-implemented, and peer-led consultation spaces on the use of MBC were launched.

### 2.6 Sustainment

Several steps were taken to ensure that clinician behavioral changes could be continued after the initial implementation was completed. The goal was to develop an efficient and cost-effective process that was self-sustaining. The quality improvement team leveraged technology platforms as much as feasible during this phase to promote scalability as new clinicians were onboarded into the system. The following sustainment activities were developed and implemented:

- Hiring criteria were adjusted to identify clinicians who are open to practicing MBC;
- The four-part training series and skills assessments were built into clinician onboarding;
- Manager training on how to supervise their clinicians in MBC was developed and conducted in April 2024;
- Consultation spaces for clinicians to receive peer feedback on MBC were set up and staffed; and
- Continuous organization-wide monitoring of patient outcomes was stood up to evaluate the durability of implementation impacts.

### 2.7 Sample

**Figure 1:**
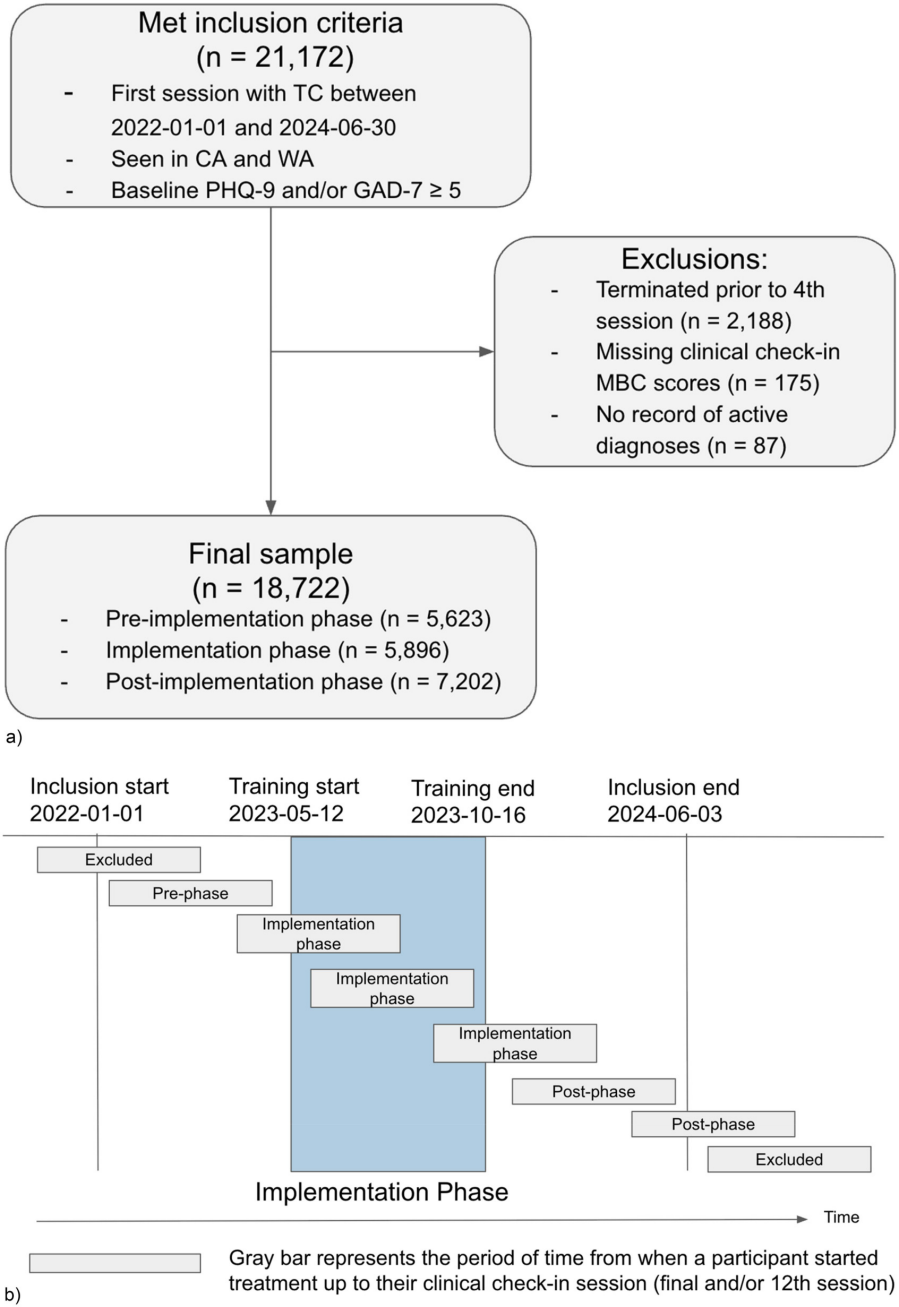
**(a)** diagram of participant inclusion and exclusion. **(b)** Participant group assignment according to the participants’ treatment start and clinical check-in dates relative to the timing of the implementation.

Clinicians were licensed master's and doctorate-level providers who were full-time employed (W2 employees) by Two Chairs. All clinicians practicing at Two Chairs were eligible for inclusion. Patients were eligible for the study if they began treatment between 01-01-2022 and 06-30-2024 in California or Washington and their baseline PHQ-9 and/or GAD-7 was ≥5 (*n* = 21,172). Patients were excluded if they terminated treatment prior to the fourth session (*n* = 2,188), were missing all MBC data between sessions 4 and 12 (*n* = 175), or had no record of an active diagnosis (*n* = 87), leaving a final analytic sample of 18,722 (88.4% of the original included population, Figure 1a).

### 2.8 Measures

Patients completed a standardized set of measures prior to every therapy session that included the PHQ-9, GAD-7, and a measure of therapeutic alliance (21, 22). The PHQ-9 is a nine-item self-report of depressive symptoms (21, 22). Items are rated on a scale ranging from 0 (not at all) to 3 (nearly every day) (21). Total scores range from 0 to 27, and cut-off scores for mild, moderate, moderately severe, and severe depressive symptoms are 5, 10, 15, and 20, respectively (21). The GAD-7 is a seven-item self-report measure of anxiety (22). Items are rated on a scale ranging from 0 (not at all) to 3 (nearly every day) (22). Total scores range from 0 to 21, and the cut-off scores for mild, moderate, and severe anxiety symptoms are 5, 10, and 15, respectively (22).

Alliance between patient and clinician was measured using a 5-item scale developed at Two Chairs that measured the following established elements of the therapeutic relationship: agreement on goals, shared understanding of tasks, and clinical bond. Items were rated on a scale of 1–5 from strongly disagree to strongly agree and averaged to create a total score ranging from 1 to 5.

### 2.9 Clinical baseline and outcomes

The first attended therapy session was defined as baseline for each patient; in the event that MBC data were not available at the first session (7% of cases), the intake assessments or second session were used, in that order. Clinical outcomes were assessed at the patient's last available session between 4 and 12, termed the “clinical check-in.” (18)

### 2.10 Phases

Patients were separated into three groups based on their session tenure relative to the timing of the project: “pre-implementation” for patients who completed their clinical check-in session prior to the launch of training on 05-12-2023, “post-implementation” for patients whose first session was after 10-16-2023, when all training activities had been completed; and “implementation” for all patients who met inclusion criteria but whose treatment didn't fall neatly into either pre or post periods (Figure 1b).

### 2.11 Implementation outcomes

To evaluate the effectiveness of the implementation in changing clinician attitudes, clinicians were asked to respond to a survey prior to the start of the project and after each module was completed. The survey asked clinicians to rate their responses to the following questions on a scale from 1 to 10 with 1 = not at all and 10 = very:

1. How important is it for you to be able to provide measurement based care to your patients?
2. How confident are you that you are able to provide measurement based care to your patients?
3. How ready are you to provide measurement based care to your patients?

### 2.12 Clinical outcomes

Three primary measures were used to measure symptom improvement at the clinical check-in session compared with baseline: percent improvement on PHQ-9, percent improvement on GAD-7, and percent improvement on PHQ-9/GAD-7 combined (PHQ-9 improvement + GAD-7 improvement)/(baseline PHQ-9 + baseline GAD-7). Adding PHQ-9 and GAD-7 scores together is a reasonable method for assessing symptoms of general affective illness, and the aggregate measure has acceptable psychometric properties (22). Percent change was selected as an outcome measure because it normalizes change across clinical and subclinical participants, and permits reasonable aggregation across these groups. To avoid large negative percent change values for patients who began with minimal initial symptoms and worsened, all outcomes were bounded between 100% improvement and 150% decline.^1^

### 2.13 Clinician behavioral outcomes

Markers of clinician behaviors associated with adherence to the MBC model were extracted from the clinical progress notes. Clinicians are required to indicate whether they discussed any measures in session and if so, which ones (PHQ-9, GAD-7, alliance). A set of binary measures (yes/no) were used to evaluate the frequency of conversations about the MBC measures across the implementation phases.

## 3 Analysis

The study population was characterized using means, standard deviations, and frequencies with statistical tests to evaluate differences between the three phases (Chi-squared tests, ANOVA) (Table 1).

**Table 1:** Baseline characteristics of patients in the three implementation periods.

Client feature	Level	Pre-phase (n = 5,624)	Implementation phase (n = 5,896)	Post-phase (n = 7,202)	p-value
Completed client profile	-	5,621 (99.9%)	5,578 (94.6%)	6,237 (86.6%)	<0.001
Age at first session	18–29 years	1,918 (34.1%)	1,694 (28.7%)	2,099 (29.1%)	<0.001
	30–49 years	2,727 (48.5%)	3,008 (51.0%)	3,551 (49.3%)	
	50–69 years	863 (15.3%)	1,018 (17.3%)	1,271 (17.6%)	
	70+ years	116 (2.1%)	176 (3.0%)	281 (3.9%)	
Gender identity	Woman	3,726 (66.3%)	3,699 (62.7%)	4,102 (57.0%)	<0.001
	Man	1,558 (27.7%)	1,546 (26.2%)	1,731 (24.0%)	
	Trans/Nonbinary/Other	303 (5.4%)	312 (5.3%)	368 (5.1%)	
	Unknown	37 (0.7%)	339 (5.7%)	1,001 (13.9%)	
Race/ethnicity	Hispanic	1,023 (18.2%)	941 (16.0%)	1,218 (16.9%)	0.001
	Black	390 (6.9%)	509 (8.6%)	594 (8.2%)	<0.001
	Asian	903 (16.1%)	816 (13.8%)	844 (11.7%)	0.001
	Other race*	725 (12.9%)	719 (12.2%)	1,086 (15.1%)	<0.001
	Unknown	111 (2.0%)	428 (7.3%)	1,164 (16.2%)	<0.001
Employment status	Full-time	3,703 (65.8%)	3,583 (60.8%)	3,855 (53.5%)	<0.001
	Caregiver	133 (2.4%)	153 (2.6%)	86 (1.2%)	
	Disabled	137 (2.4%)	143 (2.4%)	148 (2.1%)	
	Part-time	518 (9.2%)	538 (9.1%)	603 (8.4%)	
	Retired	211 (3.8%)	288 (4.9%)	364 (5.1%)	
	Student	297 (5.3%)	259 (4.4%)	278 (3.9%)	
	Unemployed	276 (4.9%)	291 (4.9%)	448 (6.2%)	
	Unknown/Other	349 (6.2%)	641 (10.9%)	1,420 (19.7%)	
Relationship status	Married	1,967 (35.0%)	2,125 (36.0%)	2,234 (31.0%)	<0.001
	Partnered	1,436 (25.5%)	1,304 (22.1%)	1,315 (18.3%)	
	Separated/Divorced/ Widowed	391 (7.0%)	425 (7.2%)	492 (6.8%)	
	Single	1,702 (30.3%)	1,608 (27.3%)	1,764 (24.5%)	
	Unknown/Other	128 (2.3%)	434 (7.4%)	1,397 (19.4%)	
Overall self-reported health	Excellent	201 (3.6%)	212 (3.6%)	303 (4.2%)	<0.001
	Very good	1,177 (20.9%)	1,099 (18.6%)	1,605 (22.3%)	
	Good/Unknown	2,501 (44.5%)	2,727 (46.3%)	3,746 (52.0%)	
	Fair	1,448 (25.7%)	1,549 (26.3%)	1,284 (17.8%)	
	Poor	297 (5.3%)	308 (5.2%)	263 (3.7%)	
Family history of mental illness	True	3,917 (69.6%)	3,902 (66.2%)	3,711 (51.5%)	<0.001
	Unknown	4 (0.1%)	320 (5.4%)	1,135 (15.8%)	
Active diagnosis categories	Depressive disorder	2,558 (45.5%)	2,891 (49.0%)	4,246 (59.0%)	<0.001
	Anxiety disorder	2,811 (50.0%)	3,213 (54.5%)	4,932 (68.5%)	<0.001
	Adjustment disorder	1,173 (20.9%)	1,313 (22.3%)	1,673 (23.2%)	0.01
	Trauma disorder	1,481 (26.3%)	1,559 (26.4%)	1,626 (22.6%)	<0.001
	Neurodevelopmental disorder	471 (8.4%)	489 (8.3%)	744 (10.3%)	<0.001
	Bipolar disorder	181 (3.2%)	172 (2.9%)	283 (3.9%)	<0.001
	OCD disorder	142 (2.5%)	140 (2.4%)	164 (2.3%)	0.69
	Alcohol disorder	76 (1.4%)	105 (1.8%)	96 (1.3%)	0.07
	Eating disorder	112 (2.0%)	92 (1.6%)	116 (1.6%)	0.15
	Substance remission disorder	73 (1.3%)	65 (1.1%)	69 (1.0%)	0.19
	Personality disorder	56 (1.0%)	72 (1.2%)	83 (1.2%)	0.50
	None of the above dx	58 (1.0%)	30 (0.5%)	9 (0.1%)	<0.001
Baseline PHQ	-	10.3 (5.3)	10.3 (5.2)	10.5 (5.3)	0.44
Baseline GAD	-	10 (4.5)	9.9 (4.6)	9.9 (4.5)	0.11
Clinical check-in session number	-	10.5 (2.6)	10.8 (2.2)	10.6 (2.4)	<0.001

### 3.1 Implementation outcomes

Scores from each assessment of clinician attitudes across the implementation period were reported as averages.

### 3.2 Clinical outcomes

The primary analysis contrasts change in anxiety and depression symptoms across the three implementation phases. Raw averages are provided for participant-level percent change in each of the three phases, stratified by baseline severity using a lower-bound cutoff score of 10 on either the PHQ-9 or GAD-7. The decision to stratify outcomes was made in order to better understand the impact of implementation across levels of severity of intake symptoms. Estimates of differences between the post-implementation and implementation phase outcomes relative to the pre-implementation outcomes were computed using linear mixed effects models. A mixed-effects model with a random intercept and indicator for treatment period was selected to account for clustering of effect by clinician. Models included random intercepts and random treatment group effects per clinician to account for clinician-level clustering of effects and unbalanced clinician contribution in the treatment group.

There are no random effects at the patient level because each patient is included using only a single observation in a single time period. These models controlled for factors known or suspected to be related to clinical outcomes, including patient diagnoses, race/ethnicity, age, gender, insurance type, state, employment status, relationship status, self-reported health, family history of mental illness, baseline PHQ-9 and GAD-7 severity categories, service state, and insurance type. The features selected for the models were chosen to minimize the amount of potential residual confounding in the contrast between the three time periods given the data available about patients. Adjustment using patient features was necessary to address potential shifts in the patient population over time. Given the very large sample sizes and the fact that parameter estimates for demographic features were not of primary importance, models were chosen to be conservative in the estimation of our primary contrast rather than parsimonious in terms of patient features. For GAD-7 and PHQ-9 outcomes, only patients whose baseline assessment was ≥5 were included. During the implementation phase, some intake data collection was changed from mandatory to optional, which resulted in decreased frequency of baseline patient information over time. Missing intake data were accounted for explicitly by using a dummy variable to encode missingness. The large transition group was included in the analysis for the purpose of providing stable estimates for these parameters that create separability between the pre-implementation period and the post-implementation with regard to these missing profile features. Given the increasing frequency of missing patient information, a sensitivity analysis was performed limited to just patients who had completed the intake data collection; the results were nearly identical.

### 3.3 Within clinician outcomes

To address a potential bias based on changes in the clinician population, a secondary analysis repeated the primary analysis of patient level-outcomes contrasting the post-phase with the pre-phase but restricted to clinicians who were present during both phases and contributed at least 10 unique patient outcomes to each phase (*n* *=* 80 clinicians). Clinician-specific slopes and intercepts were constructed based on the combination of fixed components and random clinician-specific components. To assess the relationship between baseline clinician performance and improvement across the implementation phases, the association between clinician-specific random intercepts and treatment effects were assessed with a Pearson correlation coefficient.

### 3.4 Clinical behavior change

A final analysis evaluated markers of clinician behavior change across the implementation phases. Clinician behavior change was evaluated using indicators of discussing MBC with patients in session derived from the therapy notes. Frequencies of MBC discussions reported in therapy notes from sessions 1 through 12 are shown for each of the three assessment periods along with 95% confidence intervals computed with clustered standard errors (clinician as cluster).

## 4 Results

### 4.1 Sample characteristics

#### 4.1.1 Patients

A total of *n* = 18,722 participants met requirements for analysis with 5,624, 5,896, and 7,202 in the pre-, implementation-, and post-phases, respectively (Table 1). Most participant characteristics varied significantly across the three phases (*p* < 0.05) with the exception of average baseline PHQ-9 and GAD-7 scores, which remained stable. The participants provided a diverse cross-section of treatment-seeking adults with variation across age, race, self-reported health, and employment. The most common diagnoses across all three phases were anxiety disorders, depressive disorders, and trauma disorders, respectively.

#### 4.1.2 Clinicians

A total of 755 unique clinicians contributed to the data: 361 in the pre-implementation phase, 468 during the implementation phase, and 574 post-implementation. The median number of patient observations per clinician was 13, 10, and 11 in the pre-, implementation-, and post-phases, respectively.

### 4.2 Implementation outcomes

#### 4.2.1 Clinician attitudes

Table 2 represents the average of clinician attitude scores from pre-training and after each training module. The relatively lower scores in the pre-implementation period likely represent the general lower clinician engagement in MBC prior to the initiation of the implementation. Conversely, clinicians reported high scores for importance, confidence, and readiness at all time points after the training series began.

**Table 2:** Clinician attitudes about measurement-based care assessed after each training module.

Time point	Importance	Confidence	Readiness
	Mean (SD)	Mean (SD)	Mean (SD)
Pre (*n* = 82)	6.9^a^ (2.3)	7.7 (2.1)	7.8 (1.9)
Module 1 (*n* = 241)	8.8 (1.6)	8.9 (1.4)	9.1 (1.3)
Module 2 (*n* = 208)	8.7 (1.6)	8.9 (1.2)	9.0 (1.1)
Module 3 (*n* = 198)	8.6 (1.6)	9.0 (1.2)	8.9 (1.3)
Module 4 (*n* = 480)	8.5 (1.7)	9.1 (1.1)	9.2 (1.2)

a Assessed on a scale from 1 to 10 with 1 = not at all and 10 = very.

#### 4.2.2 MBC adherence

Adherence to session-level MBC evaluation assessments in sessions 1–12 was high across all three time periods but did increase by 1.6 percentage points in the transition period and 2.4 percentage points in the post-period (pre-phase: 92.9%, implementation phase: 94.5%, post-phase: 95.3%). These differences are significant (*p* < .001), indicating improved adherence across the phases of implementation.

### 4.3 Clinical outcomes

Each individual participant contributes a percent improvement for each of the three metrics (combined PHQ-9/GAD-7, PHQ-9, and GAD-7) which reflects their relative improvement compared with their baseline assessment. An average of these individual-level percentages reflects the typical improvement seen within the group, in percentage points. An estimated difference between groups reflects an absolute difference between groups.

Table 3 characterizes the sample sizes and unadjusted clinical outcomes at the clinical check-in session across the three phases, stratified by whether the participant met the clinical cutoff for the assessment. In general, patient outcomes in the clinical group were approximately twice the size of those seen in the subclinical group across all time periods. Despite these differences, the magnitude of change associated with the pre-post comparison was a consistent approximately 5 percentage points for both clinical and subclinical groups.

**Table 3:** Raw average of percent improvement outcomes across the three phases stratified by baseline clinical status.

Measure	Outcome	Clinical			Subclinical			Overall		
		Pre	Implementation	Post	Pre	Implementation	Post	Pre	Implementation	Post
Combined MBC^a^	n	3,524	3,646	4,487	2,100	2,250	2,715	5,624	5,896	7,202
	Avg percent improvement	27.6% (26.4%,28.8%)	29.2% (28.0%,30.4%)	32.5% (31.4%,33.6%)	13.5% (11.2%,15.8%)	15.0% (12.7%,17.3%)	18.1% (16.1%,20.1%)	22.3% (21.2%,23.5%)	23.8% (22.6%,25.0%)	27.1% (26.0%,28.1%)
PHQ	n	2,832	2,958	3,706	2,124	2,254	2,690	4,956	5,212	6,396
	Avg percent improvement	29.3% (27.9%,30.7%)	30.0% (28.6%,31.3%)	33.8% (32.6%,34.9%)	16.0% (13.7%,18.4%)	18.8% (16.5%,21.1%)	21.8% (19.8%,23.9%)	23.6% (22.3%,24.9%)	25.1% (23.9%,26.4%)	28.8% (27.6%,29.9%)
GAD	n	2,674	2,742	3,367	2,534	2,667	3,281	5,208	5,409	6,648
	Avg percent improvement	31.1% (29.8%,32.4%)	33.7% (32.4%,35.1%)	36.1% (34.9%,37.3%)	15.4% (13.4%,17.4%)	16.3% (14.3%,18.3%)	20.2% (18.4%,22.0%)	23.5% (22.2%,24.7%)	25.1% (23.9%,26.4%)	28.2% (27.1%,29.3%)

a For the combined outcome, at least one of PHQ-9 or GAD-7 must be ≥10 to be considered clinical. For GAD/PHQ outcomes, the baseline value for the specific assessment must be ≥10 to be considered clinical.

Adjusted analysis was performed on the combined MBC (PHQ-9/GAD-7) percent improvement, PHQ-9 percent improvement, and GAD-7 percent improvement outcomes with results in Table 4 in absolute terms. For all three outcomes, the post-implementation phase outcomes were significantly improved over the pre-phase outcomes, with average percent improvements of approximately 5 percentage points compared to the pre-implementation period. This finding is consistent with the range seen in both the clinical and subclinical groups in unadjusted analysis. This represents a relative improvement of 23.6% on combined MBC improvement, 20.2% on PHQ-9 improvement, and 25.0% on GAD-7 improvement in the post-phase relative to the pre-phase. Modest but non-significant improvements were observed in the transition phase relative to the pre-phase.

**Table 4:** Adjusted differences in post-phase percent symptom improvement compared with the pre-phase.

Outcome	Parameter	Estimate	95% CI		*p*-value	Relative improvement
MBC percent improvement	Pre-phase average	19.5%	16.8%	22.2%	–	–
	Change in implementation phase	1.4%	−0.4%	3.2%	0.11	7.2%
	Change in post-phase	4.6%	2.8%	6.4%	<0.001	23.6%
PHQ percent improvement	Pre-phase average	23.3%	20.2%	26.4%	–	–
	Change in implementation phase	1.6%	−0.4%	3.6%	0.10	6.9%
	Change in post-phase	4.7%	2.7%	6.7%	<0.001	20.2%
GAD percent improvement	Pre-phase average	20.4%	17.5%	23.3%	–	–
	Change in implementation phase	1.6%	−0.4%	3.6%	0.12	7.8%
	Change in post-phase	5.1%	3.1%	7.1%	<0.001	25.0%

### 4.4 Within-clinician outcomes

**Figure 2:**
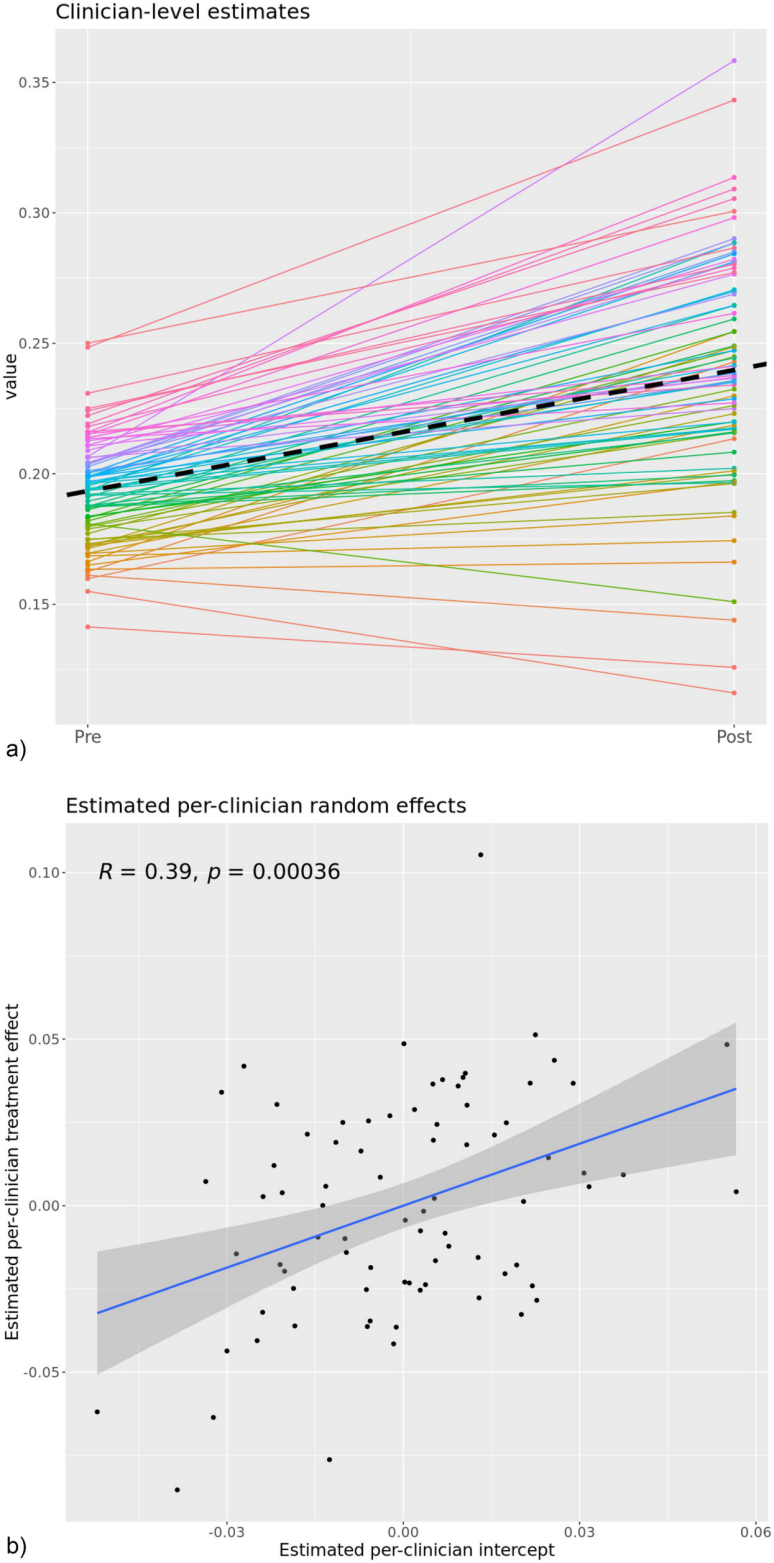
**(a)** estimated clinician-level outcomes across the pre- to post-implementation phases for *n* = 80 clinicians with sufficient pre and post data. **(b)** Clinician-specific random slopes versus random intercepts with a linear regression applied. This image represents the within-clinician change of the 80 clinicians who had sufficient data to estimate their pre vs. post phase clinical outcomes. The dashed line represents the fixed-effects slope which was significant in the model and similar in magnitude with the estimated effect in the overall model.

The secondary analysis restricted to clinicians who were present at both the pre- and post- phases reinforces the stability of the overall result; the post-phase was associated with an average improvement of 4.6 percentage points on the combined MBC outcome (*p* = 0.01) which is the same change observed in the primary analysis. Figure 2 depicts two different views of clinician-specific change from this analysis; 2a contrasts clinician-specific estimates at the pre- and post- phases based on fixed and random parameter estimates. Figure 2b plots the random treatment effect as a function of the random slope. Figure 2a demonstrates that nearly all clinicians (76 of 80; 95%) had positive clinician-specific slopes (fixed + random components), suggesting that the overall change is being driven by widespread change rather than a large change in a small number of clinicians. Figure 2b demonstrates a strong linear relationship (Pearson correlation coefficient, *r* = 0.39) between estimated random intercepts and random treatment effects such that clinicians who were already performing better than their colleagues during the pre-phase actually improved by a larger amount in the post-phase.

### 4.5 Clinical behavior change

Clinical progress notes contain structured questions about whether any MBC measures were discussed in session with the patient (and specifically whether PHQ-9, GAD-7, and/or alliance were discussed) provide a tangible measure of behavior change in clinicians. Analysis was based on 201,326 sessions across all three phases, restricted to sessions 1–12. The implementation-phase and post-phase sessions had significantly higher frequency of such discussions across all measures. In general, the frequency of discussion was higher during the implementation phase and fell slightly during the post-phase but remained substantially higher than the pre-phase (Table 5). A notable exception was discussion of therapeutic alliance, which increased across all three phases. Discussion of measures at any point during the course of care improved from 79.8% of cases during the pre-phase to 96.2% of cases during the post-phase.

**Table 5:** Frequency of discussion of MBC within session with patient as noted in progress note.

Measure	Pre-phase		Implementation phase		Post-phase	
	Frequency	95% CI	Frequency	95% CI	Frequency	95% CI
Discussed any MBC	52.0%	(48.3%,55.8%)	66.7%	(64.0%,69.4%)	62.4%	(59.8%,65.0%)
Discussed PHQ	42.9%	(39.5%,46.4%)	58.1%	(55.2%,61.0%)	55.5%	(52.8%,58.2%)
Discussed GAD	43.3%	(39.8%,46.9%)	59.0%	(56.1%,61.8%)	55.7%	(53.0%,58.5%)
Discussed Alliance	8.7%	(6.7%,10.8%)	18.2%	(15.3%,21.0%)	22.3%	(19.6%,25.1%)

## 5 Discussion

This study reports on the effect of an efficient implementation of MBC in a large-scale, technology-enabled psychotherapy practice on patient outcomes and clinician behaviors. Results showed that the implementation was associated with improvements in patients' depression and anxiety outcomes. Clinician behaviors associated with fidelity to the MBC model also increased during this time. This study suggests that an implementation completed over a relatively brief period (6 months) with primarily low-touch, self-lead training interventions can drive widespread clinical adoption of MBC and promote improvement in patient outcomes in a diverse clinical practice.

### 5.1 Improvements in depression and anxiety outcomes

The goal of implementing MBC at Two Chairs was to improve patient outcomes. Compared to patient outcomes measured prior to the project start, patient outcomes measured after the completion of the training improved by nearly 24% on a composite measure of depression and anxiety. These gains were observed among patients at both higher and lower symptom severity at baseline, and across measures of both anxiety and depression. Our analyses suggest that these gains are not attributable to changes in the composition of either the patient or clinician population. Progressive improvement in outcomes occurred across all three phases of the implementation, suggesting that these results reflect more than a transient change in provider behaviors and that these improvements may be durable. Of note, previous implementations of MBC or related practices have found that impact on patient outcomes often take up to 3–7 years to emerge and require high levels of sustained clinical oversight, suggesting that this method of implementation may be more rapid and more efficient than other approaches explored in the literature (23). These results may be attributable to combined effects of the training program, organizational alignment, technology platform, and ongoing clinical support offered.

These findings align with existing literature demonstrating the efficacy of MBC in enhancing treatment outcomes. A systematic review and meta-analysis by Scott and Lewis found that MBC was associated with significantly greater remission rates in patients with depression compared to standard care (19). The uniformity of gains across different severity levels and symptom domains underscores the transdiagnostic and transtheoretical utility of MBC and its effectiveness across diverse clinical settings (13). We observed modest and non-significant improvement during the implementation phase, which grew to robust improvements during the post-implementation phase, suggesting the benefits of the MBC implementation were sustained beyond the training period. This is consistent with findings from Lewis et al., who reported that tailored implementation of MBC led to sustained improvements in depression outcomes over time (20).

### 5.2 Evidence of change in clinician behaviors

We also found evidence that clinicians engaged in greater rates of clinical behaviors associated with adherent practice of MBC. In clinical progress notes, providers reported greater rates of discussing measures in therapy sessions (from 52.0% of sessions during the pre-implementation period to 62.4% during the post implementation period). These data help support the hypothesis that discussion of patient-reported data in session is an important factor in MBC's effectiveness (2). The evident impact of clinician-reported discussion of measures in session stands in contrast to minimal impact of merely collecting MBC data, even at very high rates. This finding highlights the limits of attending only to the organizational or technical aspects of an implementation without addressing clinical competence or leadership drivers that are required to drive meaningful and sustained change in clinical behavior.

Notably, whereas discussion of symptom measures peaked during the implementation period and dropped slightly during post-implementation, discussion of alliance started at a much lower rate at the pre-implementation period and then improved both during implementation and again at post implementation. A decrease in intervention fidelity after the cessation of implementation activities has been observed in other studies, and has been termed “voltage drop” or “program drift.” (24) Waller and Turner (2016) note that even well-intentioned clinicians may gradually drift from evidence-based practices in the absence of continued support, monitoring, or reinforcement (25). However, the continued improvement in both patient outcomes and discussion of alliance in the post-implementation period may suggest a different interpretation. The training provided during the implementation intentionally focused on the importance of alliance in promoting positive therapy outcomes and the utility of using alliance-related patient feedback in care; and further Two Chairs ceased a potentially harmful practice of using alliance as a clinician performance metric. Numerous studies suggest that sustained clinician attention to therapeutic alliance is one of the most powerful mechanisms of change in psychotherapy (14). This shift observed among providers examined in this study toward alliance focused MBC may reflect clinicians internalizing the principles of feedback informed care, moving from mechanical use of screening instruments to a more nuanced, relational integration of patient reported data (6, 14, 25). Further research is needed to disentangle the unique contributions of in-session focus on symptoms vs. alliance.

### 5.3 Improvement of individual clinicians

Among the most noteworthy findings in the current study is that of the clinicians with sufficient data to estimate changes in their pre- to post-implementation clinical outcomes, 95% showed evidence of improved outcomes (76 of 80). The magnitude of this improvement was similar to the size of the effect in the full population, providing strong support that the effect in the overall population is not simply due to a shift in the underlying clinician population but instead represents individual improvement. This analysis suggests that the implementation had a generalized positive effect on clinician performance and was not the result of large improvements for just a few providers.

Although nearly all clinicians in this subsample improved, we observed evidence of a differential impact of the implementation on groups of clinicians within this sample. As displayed in Figure 2b, the providers with the *highest* pre-implementation clinical performance (as determined by their clinical outcomes) also experienced the most improvement in clinical outcomes. This pattern is counter to what is typically observed in previous studies of MBC implementations, where gains are often most pronounced among *lower* performing clinicians (26). For example Delgadillo et al. found that routine outcome monitoring and feedback systems tend to improve outcomes primarily for clinicians with lower initial effectiveness (26). In contrast, our results align with a smaller but growing body of work suggesting that even high-performing clinicians can benefit meaningfully from feedback informed implementation efforts (27).

One possible explanation for these findings may lie in the training approach taken by Two Chairs, which utilized primarily self-directed learning on virtual modules. Given this relatively light-touch intervention, those individuals with the most motivation or innate skill may be the most able to learn and implement new skills from self-directed content. Prior studies have found that clinician engagement and motivation are key factors in mediating the impact of implementation efforts (27). If this result is replicated in other settings, systems seeking to enhance clinical quality outcomes may benefit from bifurcating training programs, with some training exercises aimed at existing high-performing staff and others aimed at medium- to low-performing staff. High-performing staff may gain organization-wide benefits when given self-directed training that is easily scalable and repeatable across cohorts.

### 5.4 Organizational strengths

There were existing organizational and leadership factors that may have enabled the success of the implementation (22). The organization had an existing robust software platform and high levels of MBC adherence. Furthermore, there was an existing commitment among clinical and company leadership to MBC as an evidence-based practice. The success of implementation may reflect strong buy-in across all organizational levels, from leadership to frontline clinicians, which helped to align strategy, ensure resource commitment, and embed MBC practices into routine workflows. This multi-level implementation strategy likely played a critical role in accelerating adoption and supporting sustained behavior change over time.

### 5.5 Limitations

Limitations of this study include its retrospective non-randomized design. This design precludes drawing clear causal inferences about the effect of implementation on patient outcomes.

Other factors, such as co-occurring organizational changes or other external events, may also have influenced the results. Future studies could use quasi-experimental designs that stagger training among staff or a truly randomized design within a set of cohorts of clinicians. A second limitation is the loss of demographic characteristics for some patients in the follow-up period, which limited our ability to control for changes in the patient population over time that may have affected the results; however, this limitation is mitigated somewhat by the results of the sensitivity analysis and inclusion of the “transition” group in the analyses.

There are also several potential threats to generalizability. First, as noted above, the organization had a strong internal commitment to MBC, a robust and proprietary software platform, and an employment model that allowed clinicians and support staff to dedicate time to training and oversight, and allowed the agency to enforce standards around MBC adherence. The agency's commitment to MBC may also have attracted clinicians who were already open to this practice, making clinician adoption of MBC practices smoother. Organizations without these features in place, including organizations where clinicians are employed on a contractual basis, may need to do more foundational work before an implementation such as the one described in this paper can be effective. Finally, it is possible that improvements in rates of discussing MBC captured in the notes could have been influenced by social desirability bias or improved diligence in documentation in the context of the training, instead of indicating clinical behavior change.

### 5.6 Implications and conclusion

This study shows that MBC implementation can be successful and sustainable, at scale, when organizations invest in and organize training and support activities in line with the best practices, including: (1) investing in organizational alignment among all key stakeholders; (2) developing intuitive, user-friendly software platforms that automate key MBC practices and provide decision support; (3) aligning messaging and setting clear expectations for clinician behaviors; (4) reinforcing the utilization of MBC principles in onboarding and ongoing staff development; and (5) proactively implementing elements designed to sustain the evidence based practice in a scalable and cost-effective way.

Beyond our primary findings, the results also illustrate the high cost of an ineffective MBC implementation. The organization's initial state, characterized by high MBC completion rates but low clinician buy-in and understanding, yielded limited impact on patient outcomes. This “implementation-in-name-only” represents a poor return on investment, incurring technological and operational costs without the corresponding clinical benefits. This distinction is critically relevant for payers and the broader shift toward value-based care. As reimbursement models increasingly focus on outcomes rather than service volume, our study suggests that to realize the value of MBC, payers and policy makers must look beyond merely mandating standardized assessments. Instead, these stakeholders should demand evidence of effective MBC implementation, including impact on clinical improvement, an outcome that aligns most closely with the goals of value-based care.

Overall, this study suggests that the structured implementation approach for MBC employed within this study was associated with improved clinical outcomes across a broad range of patients, clinical presentations, and clinicians. It also provides support for the clinical utility of MBC as an evidence-based practice that – if adequately implemented – can improve clinical outcomes in diverse clinical settings. These findings suggest a model for implementing a sustainable and effective practice of MBC.

## Data Availability

The datasets presented in this article are not readily available because these data is held by Two Chairs and therefore is not shareable. Requests to access the datasets should be directed to nforand@twochairs.com.

## Footnotes

1 Bounding the percent change outcomes to the range [-150%, 100%] resulted in minimal impact among the population meeting at least one clinical threshold at baseline ( <1% of participants were impacted by bounding). There was a larger but still small impact in the subclinical population (1.5-3.5% of participants impacted) due to the potential for larger regressions percentage-wise given lower baseline scores.
