# Supplementary material for: The impact of measurement based care at scale: examining the effects of implementation on patient outcomes and provider behaviors
Source: Front Health Serv. 2025 Nov 28;5:1659238. doi: 10.3389/frhs.2025.1659238 (PMC12698511; doi:10.3389/frhs.2025.1659238)
Supplement: Supplementary file 1 [file Table1.docx]

Supplemental Table 1: Unadjusted aggregations of other measures of symptom improvement

|  |  | **Clinical** | | | **Sub-clinical** | | | **All** | | |
| --- | --- | --- | --- | --- | --- | --- | --- | --- | --- | --- |
|  |  | **Pre** | **Transition** | **Post** | **Pre** | **Transition** | **Post** | **Pre** | **Transition** | **Post** |
| PHQ | n | 2832 | 2958 | 3706 | 2124 | 2254 | 2690 | 4956 | 5212 | 6396 |
|  | Absolute change | -4.4 (-4.6,-4.2) | -4.4 (-4.6,-4.2) | -5 (-5.2,-4.9) | -1.2 (-1.3,-1) | -1.3 (-1.5,-1.2) | -1.6 (-1.7,-1.4) | -3 (-3.1,-2.9) | -3.1 (-3.2,-2.9) | -3.6 (-3.7,-3.5) |
|  | Reliable Improvement | 48.2% (46.3%,50.0%) | 49.5% (47.7%,51.3%) | 52.6% (51.0%,54.2%) | 19.8% (18.1%,21.5%) | 20.2% (18.6%,21.9%) | 23.9% (22.3%,25.5%) | 36.0% (34.7%,37.3%) | 36.8% (35.5%,38.1%) | 40.5% (39.3%,41.7%) |
|  | Recovery | 49.6% (47.8%,51.5%) | 50.7% (48.9%,52.5%) | 54.8% (53.2%,56.4%) | - | - | - | 49.6% (47.8%,51.5%) | 50.7% (48.9%,52.5%) | 54.8% (53.2%,56.4%) |
|  | Remission | 17.0% (15.6%,18.4%) | 17.8% (16.4%,19.2%) | 20.0% (18.7%,21.3%) | 41.2% (39.1%,43.3%) | 43.8% (41.7%,45.8%) | 45.9% (44.0%,47.8%) | 27.4% (26.2%,28.6%) | 29.0% (27.8%,30.3%) | 30.9% (29.8%,32.0%) |
|  | Response | 32.3% (30.6%,34.0%) | 34.1% (32.4%,35.9%) | 36.8% (35.2%,38.3%) | - | - | - | 32.3% (30.6%,34.0%) | 34.1% (32.4%,35.9%) | 36.8% (35.2%,38.3%) |
| GAD | n | 2674 | 2742 | 3367 | 2534 | 2667 | 3281 | 5208 | 5409 | 6648 |
|  | Absolute change | -4.4 (-4.6,-4.2) | -4.8 (-5,-4.6) | -5.1 (-5.3,-4.9) | -1.1 (-1.3,-1) | -1.2 (-1.3,-1) | -1.4 (-1.5,-1.3) | -2.8 (-2.9,-2.7) | -3 (-3.1,-2.9) | -3.3 (-3.4,-3.2) |
|  | Reliable Improvement | 57.6% (55.8%,59.5%) | 61.4% (59.6%,63.2%) | 63.1% (61.5%,64.7%) | 25.6% (23.9%,27.3%) | 26.5% (24.8%,28.1%) | 29.5% (27.9%,31.1%) | 42.0% (40.7%,43.4%) | 44.2% (42.8%,45.5%) | 46.5% (45.3%,47.7%) |
|  | Recovery | 55.5% (53.7%,57.4%) | 58.8% (56.9%,60.6%) | 61.3% (59.7%,63.0%) | - | - | v | 28.5% (27.3%,29.7%) | 29.8% (28.6%,31.0%) | 31.1% (29.9%,32.2%) |
|  | Remission | 15.9% (14.5%,17.3%) | 18.3% (16.9%,19.8%) | 20.0% (18.7%,21.4%) | 38.2% (36.3%,40.1%) | 39.7% (37.9%,41.6%) | 42.0% (40.3%,43.7%) | 26.8% (25.6%,28.0%) | 28.9% (27.7%,30.1%) | 30.9% (29.8%,32.0%) |
|  | Response | 34.8% (33.0%,36.6%) | 38.4% (36.6%,40.2%) | 40.4% (38.7%,42.0%) | - | - | - | 34.8% (33.0%,36.6%) | 38.4% (36.6%,40.2%) | 40.4% (38.7%,42.0%) |

*Note.* Reliable improvement was defined as an improvement of 5pts on PHQ-9 and 4pts on the GAD-7; recovery was defined as a follow-up score <10; remission was defined as a follow-up score <5; and response was defined as a 50% or greater improvement in score from baseline.
